# Gaelic games coaches’ attitudes towards, awareness of and use of injury prevention exercise programmes

**DOI:** 10.1371/journal.pone.0351253

**Published:** 2026-06-10

**Authors:** Calvin Teahan, Enda Whyte, Siobhán O’Connor

**Affiliations:** 1 School of Health and Human Performance, Dublin City University, Dublin, Ireland; 2 Department of Health and Leisure Studies, Munster Technological University, Cork, Ireland; Università degli Studi di Milano: Universita degli Studi di Milano, ITALY

## Abstract

**Purpose:**

Gaelic games (GG) are multidirectional, evasive sports with unpredictable high-intensity activity. Lower extremity injuries are common. Injury prevention exercise programmes (IPEPs) have been introduced, effectively reducing injuries and increasing neuromuscular performance. However, the awareness and use of IPEPs are anecdotally low among coaches.

**Methods:**

Adult GG coaches (n = 342) completed an anonymous online survey, including awareness, use, and attitudes towards IPEPs and injury prevention. Frequencies and descriptive statistics were conducted, a chi-squared test was used to assess any differences in awareness and use of IPEPs, a Mann-Whitney U test was used to examine differences between groups for attitudes to injury prevention.

**Results:**

Overall, 59.5% (n = 165) of coaches reported using any form of an IPEPs, including modified or self-developed programmes, while 44.1% of coaches (n = 130) were aware of formal IPEPs. Use was statistically higher in elite coaches than non-elite (p < 0.01, phi = 0.16). No significant differences in IPEP use were observed by coach gender, team gender, or coaching education. Many coaches developed their own IPEP or altered a current IPEP, particularly participants coaching females. Coaches had a positive attitude towards injury prevention, but 40.6% stated a lack of knowledge on how to use an IPEP, with 34.5% agreeing there was no training available to teach them.

**Discussion:**

Despite generally positive attitudes towards injury prevention, awareness and implementation of formal IPEPs among GG coaches remain limited. These findings highlight the need for targeted coach education and improved dissemination strategies to enhance the adoption and effective implementation of IPEPs in GG, which is critical to mitigating the risk of injury.

## Introduction

Gaelic Games (GG) are sports native to Ireland, including Gaelic football, hurling, Ladies Gaelic football, and Camogie. GG are governed by the Gaelic Athletic Association (GAA), Ladies Gaelic Football Association (LGFA), and Camogie Association, respectively. While the GAA also governs other sports such as handball and rounders, this study focuses on the field-based, contact GG. These organisations operate through regional administrative units (e.g., county boards), which oversee local clubs and coordinate activities, with club officials (e.g., secretaries) responsible for communication and organisation at the club level. The competition spans from regional clubs (at a community level) to national inter-county events (at the elite level) [1]. GG are amateur sports, with the vast majority of coaches being volunteers [2]. Coaching commitments can be high. More than 3 in 4 GG coaches deliver at least 2–3 sessions a week [2]. It is also common for coaches to coach more than one code (one of Gaelic football, Ladies Gaelic football, hurling or Camogie) or team or level at any one time [2].

Sports participation comes with the inherent risk of injury [3]. Lower extremity injuries are the most common across all codes of GG. Injury rates vary depending on the sport and level; however, match injury rates are high in all codes (26.4–102.5 injuries/1000 hours) [4–7]. Sports-related injuries are expensive [8], and impose a substantial financial burden on the player and club. Successfully implementing widespread injury prevention practices across all GG players is crucial due to the current prevalence of injuries and the need to reduce costs for the individual player, team and society [9,10]. The coach is critical for providing safety interventions to players and is primarily responsible for injury prevention implementation [11], particularly to underage players [12] and in community level sports [13], where coaching and medical staff are lacking [14]. Given the high injury burden in GG, effective injury prevention strategies are essential.

Across a range of team sports, injury prevention exercise programmes (IPEPs) have been shown to be effective in reducing injury risk; however, their implementation in real-world settings remains inconsistent [15].

IPEPs are an effective strategy for reducing injury risk [10]. The Translating Research into Injury Prevention Practice (TRIPP) model highlights the importance of understanding the implementation context and end-user behaviour when translating injury prevention research into practice [15]. While previous research in GG has primarily focused on the development and efficacy of IPEPs, limited research has examined the later stages of the TRIPP model, particularly implementation and real-world adoption by coaches. Several GG-specific IPEPs have been developed, including the GAA15 [16], the Activate GAA Warm-up [17] the Camogie Injury Prevention Programme (CIPP) [18], and The Athletic Development and Injury Prevention Program [19]. These programmes are designed for different cohorts, with some targeting male Gaelic football and hurling players (e.g., GAA15, Activate GAA Warm-up), while others are specific to female athletes (e.g., Camogie Injury Prevention Programme and the Athletic Development and Injury Prevention Warm Up Program). This distinction is important when interpreting potential differences in awareness and use across coach and team gender. Only 3 studies have looked at the effectiveness of the GAA15 and indicated that it increased neuromuscular performance [20,21] and decreased the incidence of lower limb injuries in collegiate GG players [21], and adolescent hurlers [22]. The Activate GAA Warm-up has also been shown to enhance neuromuscular function, which could mitigate some risk factors for injury [23] While this suggests that the GAA15 and the Activate GAA Warm-up are effective, limited research has examined how these programmes are adopted and implemented in practice. Only IPEPs that coaches are aware of and use will be effective at tangibly reducing injury [11,24,25]. Although more research is needed on coaches’ awareness and use of IPEPs in GG, existing evidence suggests that awareness and use remain low. Previous research demonstrates low awareness (32%) and use (34%) of IPEPs amongst Camogie coaches [1], and similarly low use (7.7%) amongst Gaelic football coaches [26]. However, awareness was not recorded.

While previous research has examined awareness and use of IPEPs in individual GG codes, no study has comprehensively examined differences across coach gender, team gender, and multiple GG contexts. This is important to understand as coaches of female youth athletes in other sports had greater awareness and use of IPEPs than those coaching boys [27], and the various organisations governing the GG codes have different coach education pathways, which may need to be refined in the future. IPEPs can only be effective at reducing injuries if stakeholders apply them in the way that they were intended [13]. Therefore, it is critical to understand the awareness of and use of IPEPs by GG coaches.

Understanding the factors that influence IPEP adoption is essential to improving their implementation. Although IPEPs are effective, their implementation is context-dependent. GG have unique structural and coaching characteristics, which may limit the transferability of findings from other sports. Therefore, a context-specific investigation is warranted. It is essential to understand GG coaches’ attitudes to injury and injury prevention and the barriers and facilitators to successful implementation to increase the effectiveness of use, fidelity and maintaining IPEPs over time [11,28]. Even though players are these programmes’ intended end users, coaches’ attitudes affect whether or not players receive these training programmes in the first place [12,29]. Understanding and addressing barriers to IPEP implementation is important across all sports; however, this may be particularly relevant in community-based settings, where limited resources, reliance on volunteer coaches, and reduced access to medical or support staff may further constrain implementation [12]. Future developments in injury prevention are likely to occur with an improved understanding of how to use evidence-based strategies in the real world [11]. While implementation challenges are well documented in other team sports, there is limited research examining these factors within GG [1,4–7]. Only one study has looked at the attitudes of coaches, barriers and facilitators to IPEP use and coaches’ perceived ability to implement an IPEP in Camogie [1], hence, more research is needed across all codes of GG. Coach and team characteristics may influence the awareness and implementation of IPEPs. For example, previous research has suggested that coach gender and the gender of athletes may influence attitudes towards injury prevention and programme adoption, potentially due to differences in injury risk, access to resources, and coaching environments. Additionally, coaching education and level of play may impact knowledge, experience, and access to support staff, which may further influence the adoption and delivery of IPEPs. Therefore, this study aimed to: (1) determine the awareness and use of IPEPs among GG coaches; (2) examine differences based on coach gender, level of play, team gender, and coaching education; (3) explore coaches’ attitudes towards injury and injury prevention; (4) identify perceived barriers and facilitators to IPEP uses. To address these aims, the following research questions were proposed: (1) What is the level of awareness and use of IPEPs among GG coaches? (2) Do awareness and use differ based on coach gender, level of play, team gender, and coaching education? (3) What are coaches’ attitudes towards injury and injury prevention? (4) What barriers and facilitators influence the implementation of IPEPs in GG?

## Materials and methods

### Patients and study design

This study was designed and reported in accordance with the Strengthening the Reporting of Observational Studies in Epidemiology (STROBE) guidelines for cross-sectional studies (supplementary material). An anonymous online cross-sectional survey was implemented. This study was primarily descriptive in nature, with inferential analyses conducted to examine subgroup differences. The University’s Research Ethics Committee (DCUREC/2021/137) granted ethical approval prior to data collection. Participants were GG coaches recruited through county boards, club networks, and social media platforms. Invitations to participate were distributed via email and online channels. Eligible participants were those currently coaching Gaelic football, hurling, Ladies Gaelic football, or Camogie and aged 18 years or older. Participants were excluded if they were not actively coaching at the time of data collection. Participation was voluntary, and informed consent was obtained prior to survey completion. Coaching level was categorised as elite (inter-county level) or non-elite (club level).

### Instrumentation

The anonymous survey was modified from prior studies to the GG context [1,30–36]. The survey was reviewed by experts in coaching (n = 2), sport science (n = 2), and injury prevention (n = 3) to assess clarity, comprehensiveness, and appropriateness. Each question was rated on a 5-point scale, and items scoring less than 4/5 were modified or removed. The revised survey was subsequently piloted with 7 coaches representing all four GG codes to ensure clarity and relevance. Given the exploratory nature of the study and the adaptation of existing measures, formal assessment of validity was not conducted. The survey consisted of multiple sections capturing demographic information, preseason screening practices, awareness and use of IPEPs, injury prevention practices, attitudes towards injury and injury prevention, perceived barriers and facilitators, and coaches’ perceived ability to deliver IPEPs. For this study, key constructs were defined as follows: awareness referred to whether coaches had heard of or were familiar with IPEPs; use referred to the self-reported implementation of any IPEP with their team; and attitudes referred to coaches’ responses to Likert-scale statements regarding injury and injury prevention. Perceived barriers and facilitators referred to factors reported by coaches that influenced the implementation of IPEPs. A summary of the questionnaire structure is presented in Table 1. A 5-point Likert scale ranging from ‘strongly disagree’ to ‘strongly agree’ was used to assess coaches’ attitudes towards injury and injury prevention, as well as perceived barriers and facilitators to IPEP implementation.

**Table 1:** Summary of Questionnaire Structure and Number of Items per Section.

Section	Description	Number of items
Section 1	Demographic information (e.g., age, gender, coaching experience, sport, education)	8
Section 2	Preseason screening practices and access to support staff	5
Section 3	Awareness and use of IPEPs, including frequency, delivery, and reasons for non-use	9
Section 4	Injury prevention practices and frequency of use	3
Section 5	Attitudes towards injury and injury prevention	21
Section 6	Barriers and facilitators to IPEP implementation	17
Section 7	Perceived ability to deliver IPEPs	4

IPEP; injury prevention exercise programme.

### Procedures

Based upon an estimated population size of 50,000, a confidence level of 90% and a margin of error of 5%, a power analysis completed using an online calculator (Qualtrics, SAP America Inc., Seattle, WA) returned a desired sample size of 270. Every county board secretary (n = 104) and club secretary (n = 2157) in Ireland received a recruiting email with details and a survey link. The distribution of the survey to all adult coaches was requested. A reminder email was sent 3 and 6 weeks after the original email. Social media and word of mouth were used to publicise and distribute the survey. The survey was hosted online on Qualtrics (SAP America Inc., Seattle, WA) and was available from May 13 through July 15, 2022.

### Data analysis

Responses were exported to SPSS Statistics (version 27, IBM Corporation). Data were checked for errors or missing information. From the valid replies, frequencies and descriptive statistics were produced. Data distribution was assessed prior to analysis to determine appropriate statistical tests. A chi-squared test evaluated differences between the gender of the coach, levels of play, the gender of the team that they coach and between coaches who had completed a GG-specific coaching course in the use and awareness of IPEPs and if their team had access to athletic development coaches (strength and conditioning, fitness coach etc.) and medical staff (athletic therapist, physiotherapist, physical therapist etc.). Classification of effect sizes (phi) was small (0.1), medium (0.3), and large (0.5). An attitude towards injury prevention scale was created by assigning a score ranging from 1 to all “strongly disagree” replies to a value of 5 to all “strongly agree.” Negative phrases were inverted to ensure that a higher score on the scale corresponds to a more optimistic outlook towards injury prevention. Similarly, a scale of attitudes toward injury was developed from 7 statements. Internal consistency of multi-item constructs was assessed using Cronbach’s alpha, with values ≥0.70 considered acceptable. The attitudes towards injury prevention scale demonstrated acceptable internal consistency (Cronbach’s α = 0.75), while inter-item correlations for the attitudes towards injury scale were also within acceptable ranges (r = 0.2). Mann-Whitney U tests were used to determine statistically significant differences between the gender of the coach, levels of play and between coaches who had completed a GG-specific coaching course for attitudes toward injury and injury prevention. Statistical significance was set a priori at 0.05. Study data and statistical output are available at https://osf.io/fmyrh.

## Results

A total of 342 responses (254 men, 87 women and 1 non-binary/third gender) met the inclusion criteria and were included in the analysis. The number of respondents varied across variables due to item non-response; therefore, totals may differ slightly between categories. Coaches had a mean age of 48.6 ± 9.2 years and 11.7 ± 9.1 years of coaching experience. The majority of participants were non-elite coaches (90.6%), while 32 participants were classified as elite. One participant (0.3%) did not report their level of coaching and was excluded from this analysis. Additional participant characteristics are presented in Table 2.

**Table 2:** Total number and percentage of participants that coach in each Gaelic Games code.

Gaelic games code	Total % (n) (n = 342)	Elite % (n) (n = 32)	Non-elite % (n) (n = 309)
Total	–	9.4 (32)	90.6 (309)
Men’s Gaelic football only (n = 89)	26.0 (89)	18.8 (6)	26.5 (82)
Hurling only (n = 29)	8.5 (29)	9.4 (3)	8.4 (26)
Ladies Gaelic football only (n = 70)	20.5 (70)	12.5 (4)	21.4 (66)
Camogie (n = 30)	8.8 (30)	9.4 (3)	8.7 (27)
Gaelic football (both men and women) (n = 39)	11.4 (39)	15.6 (5)	11.0 (34)
Hurling and Camogie (n = 15)	4.4 (15)	12.5 (4)	3.6 (11)
Gaelic football and Hurling (n = 55)	16.1 (55)	18.8 (6)	15.9 (49)
Ladies Gaelic football and Camogie (n = 15)	4.4 (15)	3.1 (1)	4.5 (14)

%; percentage. n; number.

### Awareness and use of IPEPs

A total of 44.1% (n = 130/295) of coaches reported awareness of IPEPs, while 59.5% (n = 165/277) reported using a formal IPEP (Table 3). These proportions are based on different numbers of respondents due to item non-response. Almost half of the coaches who identify as men (47.9%) (Table 3) stated that they were aware of a specific GG IPEP, with statistically fewer women GG coaches aware (31.1%) with small effect size (*p* < 0.01, *phi* = 0.25). Similarly, awareness of IPEPs differed based on the gender of the team coached (p = 0.03, phi = 0.16) with higher awareness observed among coaches of males compared with females. There was no significant difference in coach awareness of IPEPs for those coaching at different levels of play (*p* = 0.08, *phi* = 0.12) and between coaches with or without formal GG coaching education (*p* = 0.19, *phi* = 0.08). Of the coaches that stated their awareness of a specific GG IPEP, 70.0% correctly identified one. The IPEPs that coaches were most aware of were the GAA15 (76.8%) and the Activate GAA warm-up (17.9%).

**Table 3:** Gaelic Games Coaches Awareness of, and Use of an Injury Prevention Exercise Programme and the Type utilised.

Sport/Gender	Awareness of an IPEP n = 295 % (n)	Use of an IPEP n = 277 % (n)	Injury prevention exercise programme used % (n)						
			GAA 15	Activate GAA Warm-up	Camogie Injury Prevention Programme	The Athletic Development and Injury Prevention Warm Up	Modified Injury prevention Exercise Programme	One I developed myself	Other
Total	44.1 (130)	59.5 (165)	25.9 (36)	37.4 (52)	3.6 (5)	5.0 (7)	7.2 (10)	12.2 (17)	8.6 (12)
**Gender of the coach**									
Men coaches	47.9 (109)	62.3 (139)	27.9 (31)	38.7 (43)	0.9 (1)	4.5 (5)	7.2 (8)	12.6 (14)	8.1 (9)
Women coaches	31.3 (21)	50.0 (33)	17.9 (5)	32.1 (9)	14.3 (4)	7.1 (2)	7.1 (2)	10.7 (3)	10.7 (3)
**Level of play**									
Elite	62.1 (18)	71.9 (23)	18.2 (2)	18.2 (2)	0.0 (0)	9.1 (1)	9.1 (1)	27.3 (3)	18.2 (2)
Non-elite	41.9 (111)	57.1 (148)	26.6 (34)	39.1 (50)	3.9 (5)	4.7 (6)	7.0 (9)	10.9 (14)	7.8 (10)
**Gaelic games coaching course completed**									
Yes	38.8 (40)	53.9 (55)	35.8 (19)	41.5 (22)	0.0 (0)	5.7 (3)	5.7 (3)	5.7 (3)	5.7 (3)
No	46.9 (90)	62.6 (117)	19.8 (17)	34.9 (30)	5.8 (5)	4.7 (4)	8.1 (7)	16.3 (14)	10.5 (9)
**Gender of the sport they are Coaching**									
Coaching men	48.7 (74)	61.3 (92)	32.1 (25)	41.0 (32)	0.0 (0)	6.4 (5)	7.7 (6)	7.7 (6)	5.1 (4)
Coaching women	33.3 (33)	54.6 (53)	11.4 (5)	38.6 (17)	9.1 (4)	4.5 (2)	6.8 (3)	20.5 (9)	9.1 (4)
Coaching both men and women	52.3 (23)	64.3 (27)	35.3 (6)	17.6 (3)	5.9 (1)	0.0 (0)	5.9 (1)	11.8 (2)	23.5 (4)
**Gaelic games code**									
Men’s Gaelic football	51.9 (40)	67.5 (52)	36.4 (16)	28.6 (17)	0.0 (0)	9.1 (4)	9.1 (4)	6.8 (3)	0.0 (0)
Hurling	24.0 (6)	54.2 (13)	20.0 (2)	50.0 (5)	0.0 (0)	0.0 (0)	10.0 (1)	10.0 (1)	10.0 (1)
Ladies Gaelic football	38.1 (24)	58.1 (36)	9.7 (3)	41.9 (13)	0.0 (0)	6.5 (2)	6.5 (2)	22.6 (7)	12.9 (4)
Camogie	16.6 (4)	39.1 (9)	22.2 (2)	33.3 (3)	22.2 (2)	0.0 (0)	11.1 (1)	11.1 (1)	0.0 (0)
Gaelic football (both men and women)	53.3 (16)	67.9 (19)	33.3 (4)	25.0 (3)	0.0 (0)	0.0 (0)	0.0 (0)	16.7 (2)	25.0 (3)
Hurling and Camogie	50.0 (7)	57.1 (8)	40.0 (2)	0.0 (0)	20.0 (1)	0.0 (0)	20.0 (1)	0.0 (0)	20.0 (1)
Gaelic football and Hurling	54.9 (28)	56.0 (28)	28.0 (7)	44.0 (11)	0.0 (0)	4.0 (1)	4.0 (1)	8.0 (2)	12.0 (3)
Ladies Gaelic football and Camogie	45.5 (5)	63.6 (7)	0.0 (0)	0.0 (0)	66.7 (2)	0.0 (0)	0.0 (0)	33.3 (1)	0.0 (0)

IPEP; injury prevention exercise programme. %; percentage. n; number.

Elite coaches (71.9%) had significantly higher use of IPEPs compared with non-elite (57.1%) with a small effect size (p < 0.01, phi = 0.16). However, there was no significant difference in the use of IPEPs for the gender of the coach (p = 0.20, phi = 0.11) between coaches who have or have not completed a GG coaching course (p = 0.33, phi = 0.09) and the gender of sport that they coach (p = 0.79, phi = 0.08). For the coaches that use an IPEP, the most frequently used IPEPs across all codes were the Activate GAA Warm-up (37.4%) and the GAA 15 (25.9%). In total, 12.2% of coaches reported that they had developed their own IPEP, while 7.2% reported modifying an existing IPEP (Table 4). The frequency with which IPEPs were implemented by teams is presented in Table 4. IPEPs are completed mostly at every training (45.7%) and every training and match (35.8%). Due to the structure of response options, some categories may overlap and therefore be interpreted with caution. Overall, 39.9% of coaches spent between 11 and 15 minutes delivering IPEPs with their team. For the coaches not using an IPEP, the most frequent reason was because of lack of skill set (20.6%), lack of resources (19.0%) and lack of time/too long or coaching underage (both 15.9%) (Table 4).

**Table 4:** Delivery, Frequency, and Duration of Injury Prevention Exercise Programmes in Gaelic Games and reasons for not using one.

Who is responsible for the delivery of an IPEP with your team? (n = 84) - % (n)	
Coach (me)	44.0 (37)
Coach (other)	31.0 (26)
Athletic Development Coach	16.7 (14)
Medical personnel	2.4 (2)
Player led	4.8 (4)
Self-administered outside of training	1.2 (1)
**How often are IPEPs carried out by your team (n = 162) - % (n)**	
Every training	45.7 (74)
Every match	1.2 (2)
Every training and match	35.8 (58)
One training a week	7.4 (12)
Less than one training/match a week	5.6 (9)
Completed outside of training and match time	4.3 (7)
**How long does your team spend implementing IPEP (n = 168) - % (n)**	
None	1.8 (3)
1 to 5 mins	10.7 (18)
6 to 10 mins	31.5 (53)
11 to 15 mins	39.9 (67)
16 to 20 mins	10.7 (18)
> 20 mins	5.4 (9)
**Why do you not use an IPEP with your team (n = 63) - % (n)**	
Lack of skill set to use an IPEP	20.6 (13)
Lack of resources	19.0 (12)
Coaching an underage team	15.9 (10)
Lack of time in sessions/IPEPs are too long	15.9 (10)
Never knew that they existed	7.9 (5)
Lack of confidence using an IPEP	4.8 (3)
IPEPs are too boring/repetitive	4.8 (3)
Lack of equipment	3.2 (2)
I use my own	4.8 (3)
Never thought about using one	1.6 (1)
IPEPs are poorly sequenced	1.6 (1)

%; percentage. n; number. IPEP: injury prevention exercise programme.

### Injury prevention practice

Most coaches used a warm-up (89.0%) with their team most of the time or always, along with stretching (80.5%) and a cool-down (69.4%) (Table 5). Stretching was analysed as a separate variable, although it may form part of warm-up and cool-down routines and should therefore be interpreted within this context. Injury prevention practices such as landing technique (59.3%), resistance training (50.8%) and plyometrics (42.5%) were largely, only sometimes, or never completed. Coaches rated stretching, adequate sleep, and good nutrition as the most effective injury prevention practice.

**Table 5:** Injury prevention Practice Usage and Coaches Rating of the Effectiveness of the Practice for Prevention of Injury in Gaelic Games.

	Which injury prevention practices are used with your team					How effective do you rate the following practices for injury prevention				
	Never % (n)	Sometimes % (n)	About half the time % (n)	Most of the time % (n)	Always % (n)	Total	Elite	Non-elite	Men	Women
Activation (n = 286)	29.0 (83)	24.1 (69)	8.4 (24)	21.3 (61)	17.1 (49)	6.6 ± 2.0	6.7 ± 2.4	6.6 ± 2.0	6.6 ± 2.0	6.6 ± 2.2
Active Recovery (n = 269)	14.1 (38)	23.0 (62)	13.4 (36)	31.2 (84)	18.2 (49)	7.4 ± 1.8	8.0 ± 2.2	7.4 ± 1.8	7.3 ± 1.8	7.8 ± 1.9
Adequate sleep (n = 280)	16.8 (47)	18.9 (53)	11.8 (33)	29.6 (83)	22.9 (64)	8.1 ± 1.9	8.9 ± 1.4	8.0 ± 2.0	8.0 ± 1.9	8.1 ± 2.1
Agility (n = 276)	4.0 (11)	24.3 (67)	19.6 (54)	34.4 (95)	17.8 (49)	7.8 ± 1.8	7.8 ± 2.0	7.8 ± 1.7	7.7 ± 1.7	8.1 ± 1.8
Balance (n = 274)	15.3 (42)	57.7 (116)	20.8 (57)	15.7 (43)	5.8 (16)	7.0 ± 2.4	6.3 ± 2.3	7.0 ± 2.0	6.7 ± 2.0	7.2 ± 2.1
Compression garments (n = 276)	67.4 (186)	23.2 (64)	4.7 (13)	2.5 (7)	2.2 (6)	4.3 ± 2.0	4.0 ± 2.4	4.3 ± 2.0	4.3 ± 2.0	4.3 ± 1.9
Cool down (n = 278)	4.3 (12)	16.5 (46)	9.7 (27)	19.4 (54)	50.0 (139)	7.0 ± 2.4	6.6 ± 2.7	7.0 ± 2.4	6.7 ± 2.4	7.8 ± 2.4
Core stability (n = 275)	8.7 (24)	26.2 (72)	27.6 (76)	27.6 (76)	9.8 (27)	7.8 ± 1.9	7.9 ± 1.4	7.8 ± 1.9	7.6 ± 1.9	8.4 ± 1.5
Cryotherapy (n = 277)	69.3 (192)	23.5 (65)	5.1 (14)	1.4 (4)	0.7 (2)	4.7 ± 2.4	4.8 ± 2.6	4.6 ± 2.4	4.7 ± 2.5	4.5 ± 2.3
Education (n = 277)	31.4 (87)	38.6 (107)	13.4 (37)	10.5 (29)	6.1 (17)	7.1 ± 2.5	8.0 ± 2.1	7.0 ± 2.5	7.0 ± 2.5	7.5 ± 2.5
Endurance (n = 275)	11.6 (32)	35.6 (98)	21.1 (58)	22.9 (63)	8.7 (24)	6.3 ± 2.1	7.5 ± 2.0	6.2 ± 2.1	6.3 ± 2.1	6.4 ± 2.2
Foam rolling (n = 277)	30.1 (103)	35.0 (97)	7.6 (21)	10.1 (28)	10.1 (28)	6.0 ± 2.5	6.3 ± 2.3	6.0 ± 2.5	5.9 ± 2.5	6.5 ± 2.5
Good nutrition (n = 278)	12.2 (34)	23.7 (66)	16.2 (45)	24.8 (69)	18.7 (64)	8.2 ± 1.8	9.1 ± 1.1	8.1 ± 1.9	8.2 ± 1.9	8.5 ± 1.7
Knowledge of the rules (n = 267)	11.50 (21	21.9 (61)	19.1 (53)	30.2 (84)	17.3 (48)	6.3 ± 2.8	6.6 ± 2.9	6.3 ± 2.8	5.9 ± 2.7	7.8 ± 2.7
Load management (n = 276)	15.2 (41)	17.3 (48)	22.4 (62)	23.8 (66)	21.3 (59)	8.0 ± 1.8	8.7 ± 1.8	7.9 ± 1.8	8.1 ± 1.8	7.5 ± 2.0
Massage (n = 281)	50.7 (141)	30.9 (86)	6.8 (19)	8.6 (24)	2.9 (8)	5.7 ± 2.2	5.9 ± 2.7	5.6 ± 2.1	5.5 ± 2.1	6.1 ± 2.4
Orthoses (n = 275)	64.7 (178)	26.5 (73)	4.7 (13)	2.2 (6)	1.8 (5)	5.1 ± 2.5	4.7 ± 2.2	5.1 ± 2.5	5.1 ± 2.5	5.1 ± 2.4
Plyometrics (n = 273)	12.5 (34)	30.0 (82)	27.1 (74)	17.2 (47)	13.2 (36)	7.3 ± 1.9	7.7 ± 1.8	7.3 ± 1.9	7.2 ± 1.9	7.6 ± 1.9
Recovery boots (n = 274)	76.6 (210)	16.4 (45)	4.7 (13)	1.5 (4)	0.7 (2)	4.3 ± 2.4	4.7 ± 2.7	4.3 ± 2.3	4.3 ± 2.3	4.4 ± 2.5
Resistance training (n = 276)	17.8 (49)	33.0 (91)	18.1 (50)	21.4 (59)	9.8 (27)	7.0 ± 2.0	8.2 ± 1.8	6.9 ± 2.0	7.0 ± 2.0	5.1 ± 2.1
Speed training (n = 277)	4.0 (11)	21.7 (60)	23.1 (64)	33.9 (94)	17.3 (48)	7.3 ± 2.0	8.4 ± 1.6	7.2 ± 1.9	7.3 ± 2.0	7.2 ± 2.1
Stretching (n = 277)	2.9 (8)	10.8 (30)	5.8 (16)	22.0 (61)	58.5 (162)	8.2 ± 2.0	7.8 ± 2.3	8.3 ± 2.0	8.0 ± 2.1	9.0 ± 1.6
Taping (n = 273)	32.6 (89)	39.6 (108)	12.8 (35)	9.5 (26)	5.5 (15)	5.2 ± 2.3	5.1 ± 2.7	5.2 ± 2.3	5.1 ± 2.3	5.7 ± 2.5
Training of functional movements (n = 278)	14.9 (41)	30.9 (85)	27.3 (75)	18.2 (50)	8.7 (24)	7.6 ± 2.0	8.0 ± 1.9	7.5 ± 2.0	7.6 ± 1.9	7.5 ± 2.4
Landing technique (n = 275)	22.9 (63)	36.4 (100)	21.8 (60)	11.3 (31)	7.6 (21)	7.3 ± 2.0	7.6 ± 1.8	7.3 ± 2.0	7.2 ± 1.9	7.6 ± 2.2
Proper sports technique (n = 274)	11.7 (32)	19.0 (52)	21.9 (60)	25.9 (71)	21.5 (59)	7.7 ± 1.9	7.9 ± 1.9	7.7 ± 1.9	7.6 ± 1.8	8.0 ± 2.2
Warmup (n = 273)	0.4 (1)	5.9 (16)	4.8 (13)	16.5 (45)	72.5 (198)	8.0 ± 2.0	7.7 ± 2.0	8.0 ± 2.0	7.7 ± 1.9	8.9 ± 1.7

%; percentage. n; number.

### Attitudes towards injury and injury prevention, and perceived barriers and facilitators

A total of 93.1% (n = 240) of coaches agreed or strongly agreed that injuries can shorten a player’s career, while 94.2% (n = 244) agreed or strongly agreed that injuries can cause problems later in life (Table 6). Additionally, 75% (n = 193) of coaches agreed or strongly agreed that GG players are at high risk of injury. However, only 36.2% (n = 94) agreed or strongly agreed that injuries were an issue within their own team. Women coaches demonstrated significantly higher scores on the attitudes towards injury scale (median = 19), indicating a more negative perception of injury compared with men (median = 18), with a medium effect size (r = 0.43, *p* = 0.01). There were no significant differences in attitudes towards injury for the level of play (r = 0.03, *p* = 0.47), between coaches who have completed a GG coaching course (r = 0.05, *p* = 0.35) and the gender of sport that they coach (r = 0.27, *p* = 0.12).

**Table 6:** Coaches’ Attitudes Towards Injury and IPEPs and Barriers and Facilitators to Successful Use of IPEPs.

	Strongly disagree % (n)	Disagree % (n)	Neither agree or disagree % (n)	Agree % (n)	Strongly agree % (n)
**Negative statements to injury and injury prevention**					
Injuries are an issue with my team (n = 260)	9.6 (25)	25.8 (67)	28.5 (74)	28.5 (74)	7.7 (20)
Injuries can shorten a player’s career (n = 258)	3.9 (10)	1.2 (3)	1.9 (5)	32.6 (84)	60.5 (156)
Injuries can cause physical problems later in life (n = 259)	2.7 (7)	0.0 (0)	3.1 (8)	41.3 (107)	52.9 (137)
Injuries have a negative impact on team performance (n = 257)	1.6 (4)	1.6 (4)	9.3 (24)	40.1 (103)	47.5 (122)
Gaelic games players are at a high risk of suffering an injury (n = 260)	2.3 (6)	1.5 (4)	21.9 (57)	46.9 (122)	27.3 (71)
IPEPs cost too much (n = 259)	16.2 (42)	27.0 (70)	42.9 (111)	12.7 (33)	1.2 (3)
IPEPs takes up too much training time away from necessary tasks (n = 260)	21.5 (56)	42.7 (111)	28.5 (74)	6.5 (17)	0.8 (2)
**Positive statements to injury and injury prevention**					
It’s important for coaches to have current knowledge of IPEPs (n = 259)	1.5 (4)	0.8 (2)	1.9 (5)	47.1 (122)	48.6 (126)
It’s important for players to have current knowledge of IPEPs (n = 259)	1.5 (4)	0.0 (0)	1.5 (4)	51.7 (134)	45.2 (117)
Injury prevention is important during training sessions (n = 258)	0.8 (2)	0.8 (2)	3.1 (8)	52.3 (135)	43.0 (111)
Activities included in IPEPs are relevant and beneficial to players (n = 259)	0.8 (2)	0.4 (1)	4.6 (12)	51.4 (133)	42.9 (111)
I believe that using an IPEP will reduce the number of injuries for my team (n = 258)	0.8 (2)	0.4 (1)	7.4 (19)	50.4 (130)	41.1 (106)
I believe that injuries are preventable (n = 258)	1.9 (5)	8.9 (23)	25.2 (65)	47.7 (123)	16.3 (42)
Exercises which have been shown to prevent injuries should be performed by Gaelic games players (n = 259)	0.0 (0)	0.0 (0)	4.2 (11)	51.7 (134)	44.0 (114)
Exercises to prevent injuries should be varied and progressed over time (n = 258)	0.0 (0)	0.0 (0)	7.4 (19)	60.1 (155)	32.6 (84)
**Attitudes on Safety Behaviours**					
I believe that it is safe to play with injuries (n = 259)	41.3 (107)	41.7 (108)	11.6 (30)	3.1 (8)	2.3 (6)
I am willing to let my players play with injuries (n = 260)	45.0 (117)	35.8 (93)	11.2 (29)	6.9 (18)	1.2 (3)
I admire Gaelic games players who continue to play when injured (n = 259)	37.8 (98)	39.8 (103)	15.4 (40)	5.4 (14)	1.5 (4)
**Perceived behavioural control factors relating to the level of support received, or expected to be received, if the player had been injured or were to be injured**					
I believe that players should be fully rehabilitated before playing again after they have suffered an injury (n = 259)	5.4 (14)	6.2 (16)	11.2 (29)	35.5 (92)	41.7 (108)
I support players when they are injured (n = 259)	2.3 (6)	1.5 (4)	3.1 (8)	51.4 (133)	41.7 (108)
The club assist players with medical issues (n = 260)	5.0 (13)	6.5 (17)	17.7 (46)	45.8 (119)	25.0 (65)
**Facilitators to IPEP use**					
Observing elite teams (n = 237)	1.7 (4)	3.8 (9)	14.8 (35)	56.1 (133)	23.6 (56)
Better resources (n = 238)	1.7 (4)	3.4 (8)	10.1 (24)	57.6 (137)	27.3 (65)
More training in the delivery (n = 237)	1.7 (4)	1.7 (4)	11.4 (27)	58.2 (138)	27.0 (64)
The introduction of a ball or skills (n = 236)	1.3 (3)	4.7 (11)	12.7 (30)	52.5 (124)	28.8 (68)
A player in my team having a serious injury (n = 234)	3.4 (8)	11.5 (27)	21.4 (50)	48.3 (113)	15.4 (36)
A player would run faster (n = 235)	5.5 (13)	12.3 (29)	27.2 (64)	40.0 (94)	14.9 (35)
A player would jump higher (n = 234)	6.4 (15)	16.2 (38)	28.6 (67)	35.0 (82)	13.7 (32)
A player would have fewer risk factors for injury (n = 235)	2.1 (5)	3.4 (8)	10.6 (25)	51.5 (121)	32.3 (76)
I would feel comfortable leading IPEP (n = 234)	2.1 (5)	13.2 (31)	17.1 (40)	40.2 (94)	27.4 (64)
I would feel comfortable with a player leading IPEP (n = 234)	3.0 (7)	13.2 (31)	16.7 (39)	52.6 (123)	14.5 (34)
I would feel comfortable with another coach leading IPEP (n = 233)	1.3 (3)	5.2 (12)	12.4 (29)	52.8 (123)	28.3 (66)
I would feel comfortable with my athletic development coach leading IPEP (n = 234)	0.0 (0)	1.3 (3)	7.3 (17)	46.2 (108)	45.3 (106)
I would feel comfortable with my ATT/Physio Leading IPEP (n = 231)	0.0 (0)	1.3 (3)	11.3 (26)	48.5 (112)	39.0 (90)
**Barriers to IPEP use**					
My teams training sessions are not long enough (n = 249)	14.5 (36)	41.0 (102)	22.5 (56)	18.5 (46)	3.6 (9)
IPEP is too long (n = 248)	13.3 (33)	35.9 (89)	37.9 (94)	12.9 (32)	0.0 (0)
Exercises too difficult (n = 249)	18.1 (45)	48.6 (121)	29.3 (73)	3.6 (9)	0.4 (1)
Exercises are boring (n = 247)	11.7 (29)	38.1 (94)	33.2 (82)	15.0 (37)	2.0 (5)
The programme is too rigid (n = 248)	10.5 (26)	31.9 (79)	44.4 (110)	11.7 (29)	1.6 (4)
I am not motivated enough (n = 248)	19.0 (47)	41.5 (103)	29.0 (72)	10.1 (25)	0.4 (1)
There is no ball/hurley involved (n = 247)	14.6 (36)	30.4 (75)	31.2 (77)	21.5 (53)	2.4 (6)
There is no training available to teach me (n = 246)	14.2 (35)	30.5 (75)	20.7 (51)	27.6 (68)	6.9 (17)
I do not believe that using an IPEP will reduce injuries (n = 246)	34.1 (84)	47.6 (117)	10.2 (25)	4.9 (12)	3.3 (8)

%; percentage. n; number. IPEP; injury prevention exercise programmes.

GG coaches agreed that it is important for both coaches (95.7%) and players (96.9%) to have current knowledge of IPEPs. A total of 91.5% of coaches agreed that using an IPEP will reduce the number of injuries to their team. There were no significant differences in attitudes towards injury for the gender of the coach (r = 0.14, *p* = 0.13), the level of play (r = 0.07, *p* = 0.29) between coaches who have completed a GG coaching course (r = 0.18, *p* = 0.09) and the gender of sport that they coach (r = 0.13, *p* = 0.34). More training in delivering IPEPS (85.2%) and better resources (84.9%) were the greatest facilitators of using IPEPs. A total of 40.6% of coaches reported insufficient knowledge to use IPEPs, while 34.5% agreed that there was no training available to teach them (Table 6).

### Access to athletic development coaches and medical personnel

Just under half of coaches had access to an athletic development coach with their team (47.7%). A significantly greater number of men coaches (51.6%) had access compared with women coaches (35.6%), with a small effect size (*p* = 0.04, *phi* = 0.17). Similarly, elite coaches (87.5%) also had greater access to athletic development coaches compared with non-elite coaches (43.4%) (*p* < 0.01, *phi* = 0.26). There was also a significant difference between access to athletic development coaches and the gender of the sport that they coach (*p* < 0.01, *phi* = 0.22). Post-hoc comparisons found that participants coaching women only (32.8%) had significantly less access to athletic development coaches compared with those coaching men only (55.8%) (r = 0.22, *p* < 0.01) and those coaching both men and women (53.7%) (r = 0.20, *p* = 0.01). There was no significant difference in access between coaches who have completed a GG coaching course (*p* = 0.11, *phi* = 0.11). Access to medical personnel is described in Table 7. Overall, 34.7% of teams had access to medical personnel at least every match. Almost half of participants coaching men’s teams only (56.0%) stated that they had access to medical personnel at least every match. However, 57.4% of coaches that coached females only stated that they never had access to medical personnel.

**Table 7:** Access to medical personnel.

	Every training and match % (n)	One training a week and every match % (n)	Every match only % (n)	Championship match only % (n)	Every training only % (n)	One training a week only % (n)	Occasional % (n)	Never % (n)
Total (n = 341)	10.6 (36)	5.6 (19)	18.5 (63)	8.5 (29)	0.6 (2)	0.6 (2)	18.2 (62)	37.4 (128)
**Gender**								
Men coaches (n = 254)	11.4 (29)	6.3 (16)	21.7 (55)	8.3 (21)	0.8 (2)	0.8 (2)	16.5 (42)	34.3 (87)
Women coaches (n = 86)	7.0 (6)	3.5 (3)	9.3 (8)	9.3 (8)	0.0 (0)	0.0 (0)	23.3 (20)	47.7 (41)
**Gaelic games code**								
Gaelic football (n = 89)	12.4 (11)	4.5 (4)	30.3 (27)	6.7 (6)	1.1 (1)	1.1 (1)	23.6 (21)	20.2 (18)
Hurling (n = 29)	27.6 (8)	10.3 (3)	24.1 (7)	10.3 (3)	0.0 (0)	0.0 (0)	13.8 (4)	13.8 (4)
Ladies Gaelic football (n = 70)	2.9 (2)	0.0 (0)	5.7 (4)	8.6 (6)	0.0 (0)	0.0 (0)	25.7 (18)	57.1 (40)
Camogie (n = 29)	13.8 (4)	3.4 (1)	6.9 (2)	10.3 (3)	0.0 (0)	0.0 (0)	13.8 (4)	51.7 (15)
Both men and women Gaelic football (n = 29)	12.8 (5)	7.7 (3)	20.5 (8)	2.6 (1)	0.0 (0)	0.0 (0)	17.99 (7)	38.5 (15)
Both hurling and Camogie (n = 15)	20.0 (3)	6.7 (1)	20.0 (3)	13.3 (2)	0.0 (0)	0.0 (0)	6.7 (1)	33.3 (5)
Both male codes (n = 55)	5.5 (3)	10.9 (6)	18.2 (10)	12.7 (7)	1.8 (1)	1.8 (1)	12.7 (7)	36.4 (20)
Both female codes (n = 15)	0.0 (0)	6.7 (1)	13.3 (2)	6.7 (1)	0.0 (0)	0.0 (0)	0.0 (0)	73.3 (11)
**Level of play**								
Elite (n = 32)	40.6 (13)	9.4 (3)	28.1 (9)	6.3 (2)	3.1 (1)	0.0 (0)	6.3 (2)	6.3 (2)
Non-Elite (n = 308)	7.5 (23)	5.2 (16)	17.2 (53)	8.8 (27)	0.3 (1)	0.6 (2)	19.5 (60)	40.9 (126)
**Gender of the sport they are coaching**								
Men only (n = 172)	12.8 (22)	7.6 (13)	25.6 (44)	8.7 (15)	1.2 (2)	1.2 (2)	18.6 (32)	24.4 (42)
Women only (n = 115)	5.2 (6)	1.7 (2)	7.0 (8)	9.6 (11)	0.0 (0)	0.0 (0)	19.1 (22)	57.4 (66)
Both men and women (n = 54)	14.8 (8)	7.4 (4)	20.4 (11)	5.6 (3)	0.0 (0)	0.0 (0)	14.8 (8)	37.0 (20)
**Gaelic Games coaching course completed**								
Yes (n = 117)	6.0 (7)	4.3 (5)	15.4 (18)	12.0 (14)	0.0 (0)	0.8 (1)	20.5 (24)	41.0 (48)
No (n = 200)	12.9 (29)	6.3 (14)	20.1 (45)	6.7 (15)	0.9 (2)	0.4 (1)	6.3 (14)	35.7 (80)

%; percentage. n; number.

### Preseason screening

A total of 23.2% of coaches conducted preseason screening with their team to evaluate the risk of injury. A greater proportion of men coaches (27.2%) reported conducting preseason screening compared to women coaches (10.5%) with small effect size (*p* < 0.01, phi = 0.21). A greater proportion of elite coaches (65.0%) conducted preseason screening compared to non-elite coaches (18.4%) with medium effect size (*p* < 0.01, *phi* = 0.33). There was a significant difference in coaches that conducted preseason screening and the gender of the sport they coached (*p* = 0.01, *phi* = 0.23). Post-hoc comparisons found that those coaching women only (10.3%) conducted significantly less preseason screening compared with participants coaching men only (30.8%) (r = 0.23, *p* < 0.01) and participants coaching both men and women (25.9%) (r = 0.20, *p* = 0.01). There was no significant difference in preseason screening between coaches who have completed a GG coaching course (*p* = 0.10, *phi* = 0.12).

## Discussion

This study aimed to understand GG coaches’ awareness of, use and perceived ability to implement an IPEP with their team. Further, we aimed to investigate coaches’ attitudes towards injury and injury prevention and the barriers and facilitators to successful IPEP implementation. Overall, 44.1% of coaches reported awareness of formal IPEPs, with 70.0% of these able to correctly identify a programme. Therefore, only 34.8% of all coaches could both recognise and name an IPEP. This is consistent with previous findings in Camogie coaches (32%) [1], Canadian high school rugby coaches (27%) [37], and youth male soccer coaches (16%) [38]. However, this is lower than coaches’ awareness in youth soccer (58–65%) [39,40], youth rugby (75%) [41], high school basketball and soccer (52%) [42], and European amateur soccer (42.6%) [35]. Low awareness is concerning as awareness is the critical first step in the implementation process [13]. Despite 36.2% of coaches recognising injuries as an issue within their team, many remain unaware of available IPEPs.

The cost of an injury can be burdensome, with the mean cost of men’s GG claims being €1158.40 [43], and the mean cost of ladies Gaelic football claims being €663.30 [44], with claims in both increasing annually. Previously it was reported that 63.8% of claims made were for lower limb injuries [44], which have been the focus of IPEP, with the GAA15 proven to reduce lower limb injuries [21,22]. Therefore, organisational-level strategies are needed to increase awareness, particularly in community-based GG where coaches drive implementation [13]. There was a significant difference in awareness between the gender of the coaches and the gender of the sport they coached with women coaches, and those coaching female GG were less aware of IPEP. Previous research has demonstrated greater exercise fidelity among female athletes, which has been attributed to higher perceived injury severity in female athletes [27]. This heightened perception of risk may encourage greater engagement with injury prevention strategies and could, in turn, contribute to increased awareness of IPEPs in female sporting contexts. However, this was not the case in GG. The female GG organisations need to target coaches and increase awareness of IPEPs with women coaches and coaches of female teams as female players may have less access to medical care [45] and discrepancies in funding between male and female sports [46]. Therefore, coaches may have increased responsibility to deliver IPEPs with their team.

A total of 59.5% (n = 165/277) of coaches reported using any form of IPEP. This proportion reflects responses from a subset of participants and should be interpreted in the context of varying response rates across questions. This is almost double what was reported in Camogie (34%) [1], and much more than in Gaelic football (7.2%) [26]. However, it must be noted that the Gaelic football study had a small sample size and only included 2 counties [26]. Thus, while the widespread use of IPEPs is still relatively low, it has increased in recent years. Coaches with elite teams utilised IPEPs more than non-elite coaches, which may be explained by the greater access to athletic development coaches and medical personnel who have been identified as essential programme implementers in elite sports [47]. Therefore, clubs and organisations need to educate coaches on IPEP, particularly coaches of non-elite teams, as coaches are in a distinctive position to encourage injury prevention, safe play, and make rapid choices on injury management [48], particularly in amateur sports such as GG. Coaches identified lack of knowledge and resources as primary barriers to IPEP use. Although IPEPs are freely available, additional education and practical support are required to improve confidence in their delivery. Practical workshops have been shown to enhance implementation, adherence, and perceived competence, suggesting that structured coach education should be prioritised across GG [1,2]. Previous research has shown in Camogie coaches that an injury prevention workshop with both theory and practical elements enhanced coaches’ attitudes towards injury prevention, increased the implementation and maintenance of an IPEP and enhanced their perceived ability in their skill to conduct the IPEP [49]. Similarly, participation in a coaching workshop on FIFA 11 + training is reported to have increased programme adherence compared to only giving out instructional materials [50]. Injury prevention education is now embedded in Camogie coaches’ education, but educational injury prevention workshops should be made mandatory for all GG codes as they are effective, and coaches would like to attend them.

Although IPEPs are designed to be implemented at every training session and match, only 35.8% of teams reported using them as intended. Additionally, 44% of coaches allocated less than 10 minutes to IPEPs, below recommended durations. As programme effectiveness depends on correct implementation, improving fidelity is essential [51]. Future studies should determine the minimal dose required for IPEPs, but until then, IPEPs should be used every training and match as directed. Coaches also agreed (92.7%) that IPEPs must be varied and progress over time. These elements were previously thought to be crucial for inspiring players, preventing monotony, and tailoring the exercises to individual players’ various skill levels [52]. The Activate has some variation of the exercise, which may explain why it was the most used IPEP amongst GG coaches. However, the GAA15 has no variation or progressions. Coaches must be invited to contribute to the creation of the IPEPs from the start [53]. Further qualitative research is needed to investigate why so many coaches create their own IPEPs, particularly in ladies’ GG. Understanding why coaches modify IPEPs is important, as these changes may affect programme efficacy [52]. It was previously found that lower compliance or IPEP fidelity was associated with an increased injury rate in male athletes [54–56]. However, allowing coaches autonomy might increase coach compliance, particularly their willingness to adopt the IPEPs [57]. Although no previous research has looked at programme fidelity in GG IPEPs, based on the current research in other sports, poor programme fidelity would likely lead to less-than-optimal programme effectiveness in GG. Therefore, the GG organisations must include GG coaches in the design phase of IPEPs which will aid in coach motivation to use the programmes as designed.

Warm-up (89.0%), stretching (80.5%), and cool-down (69.4%) were the most commonly used practices. This is similar to basketball and Canadian rugby coaches, where 95.9% and 85% of coaches implement a warm-up, respectively [37,58]. While this indicates a willingness to adopt injury prevention practices, some commonly used practices are not strongly supported by evidence. Resistance training has been demonstrated to reduce injuries by 69%, compared to stretching’s 4% reduction [59]. These findings indicated the need for improved education on evidence-based practices, which will lead to a greater injury risk reduction for GG players. The results indicate a general willingness to employ injury prevention practice. However, there needs to be both a top-down and bottom-up approach, where researchers listen to the stakeholders and contextual factors to increase adoption. This aligns with the later stages of the TRIPP model, which emphasise the importance of incorporating contextual factors and stakeholder engagement to improve the implementation of injury prevention strategies [13]. The GG IPEPs themselves may need to be adapted to meet the needs of coaches, but also coaches need to be educated on the essential aspects of injury prevention if there is going to be an effective injury risk reduction in GG.

It takes the engagement of many vital stakeholders to improve IPEP uptake and implementation in real community sports settings [53]. It is also critical to understand how stakeholders feel about injury prevention and how it fits into their lifestyles and the game’s culture [51]. Therefore, it is crucial to understand GG coaches’ attitudes to injury prevention. Overall 93.1% of GG coaches agreed that injuries can shorten a player’s career, and 74.2% of coaches agreed GG players are at a high risk of injury. Similarly, 87.6% of coaches agreed that injuries can have a negative impact on team performance. Research has shown that injuries negatively affect team success in professional soccer [60], professional rugby [61], and Australian rules football [62]. Most coaches (95.7%, n = 259)) agreed it is important to have current knowledge of IPEPs and 95.3% agreed that injury prevention is important during training. Coaches understand the negative impacts that injury can have, and there is potential for encouraging the use of an IPEP. Therefore, it is paramount that organisations emphasise educating these volunteer coaches on how to deliver an IPEP to their team, so that coaches understand the risks involved with GG and the importance for coaches to have current knowledge of IPEPs.

For evidence-based IPEPs to have a meaningful impact on preventing injuries, they must be widely and consistently implemented [11]. Understanding the barriers and facilitators in a GG setting is critical to IPEP implementation [15]. Coaches stated that having an athletic development coach (91.5%) or an athletic therapist/physiotherapist (87.5%) lead their team in IPEPs were the greatest facilitators of using an IPEP. However, most teams have no access to athletic development coaches or medical personnel (Table 7). Key barriers included lack of knowledge (40.6%), lack of training (34.5%), and insufficient expertise within teams (31.3%). Despite IPEPs being designed for use by non-specialist coaches [13,63], their uptake remains limited. This highlights the need for structured dissemination strategies and targeted education to improve adoption and implementation. The transmission of information about an invention (IPEP) to a potential user without a structured dissemination campaign relies significantly on mass media and human contacts [64]. Therefore, organisations need to adopt an injury prevention strategy to maximise awareness, use and fidelity of IPEPs.

### Limitations

This study was the first to investigate the implementation of IPEPs across all GG codes and coaches’ attitudes towards injury prevention. Several limitations related to sampling and data collection should be considered. The use of an online survey may have introduced self-selection bias, whereby coaches with a greater interest in injury prevention were more likely to participate. Additionally, the representativeness of the sample across different competition levels and regions is unknown, which may limit the generalisability of the findings. Non-response bias may also have influenced the results, as not all participants completed every question. Although internal consistency was assessed for multi-item constructs, further psychometric evaluation of the survey instrument was not undertaken. In particular, test–retest reliability and construct validity were not examined, which may limit the robustness of the measures used. Another limitation of this study relates to the structure of the survey response options for IPEP frequency. Some response categories may have overlapped. This may limit the precision and interpretability of these findings. Stretching was examined as a distinct component of injury prevention; however, it is often incorporated within broader warm-up and cool-down routines. This may limit the independence of these findings and should be considered when interpreting the results. Respondents’ answers to the survey’s Likert scale questions may have been influenced by central tendency bias, acquiescence bias, or social desirability bias. Gaining a more profound knowledge of end-user attitudes and contextual challenges in adopting IPEP may be aided by additional qualitative research. Although perceived ability to deliver IPEPs is an important factor in implementation, it was not examined in detail in the current study and should be explored in future research.

## Conclusion

Although efficacious IPEPs have been developed in GG, injury prevention requires much more than an effective programme. For any change in injury rates, the efficacious IPEPs must be accepted, used and maintained with high fidelity by the end users. However, less than half of GG coaches were aware that GG IPEPs existed, and just over a third were using a specific GG IPEP. Where IPEPs were used, fidelity was low. There is a potential for organisations and clubs to encourage coaches to use IPEPs and enhance coaches’ skills in delivering IPEPs as GG coaches acknowledge the negative impact injuries can have, and they have a positive attitude towards injury prevention. This could be achieved by acknowledging some barriers and facilitators identified by GG coaches, such as more training in delivering IPEPs and better resources, such as access to medical personnel and coaches. Similarly, organisations need to include coaches in the development of injury prevention strategies. A greater emphasis must be placed on educating coaches across all codes of GG, particularly with coaches involved with female teams.
